# Effective Perturbations by Small-Molecule Modulators on Voltage-Dependent Hysteresis of Transmembrane Ionic Currents

**DOI:** 10.3390/ijms23169453

**Published:** 2022-08-21

**Authors:** Sheng-Nan Wu, Chao-Liang Wu, Hsin-Yen Cho, Chi-Wu Chiang

**Affiliations:** 1 Department of Physiology, National Cheng Kung University Medical College, Tainan 70101, Taiwan; 2 Institute of Basic Medical Sciences, National Cheng Kung University Medical College, Tainan 70101, Taiwan; 3 Department of Post-Baccalaureate Medicine, National Sun Yat-sen University, Kaohsiung 804201, Taiwan; 4 Department of Medical Research, Ditmanson Medical Foundation Chia-Yi Christian Hospital, Chiayi City 60002, Taiwan; 5 Institute of Molecular Medicine, College of Medicine, National Cheng Kung University, Tainan 70101, Taiwan

**Keywords:** voltage-dependent hysteresis, hyperpolarization-activated cation current, *erg*-mediated K^+^ current, M-type K^+^ current, L-type Ca^2+^ current, persistent Na^+^ current, small-molecule modulators

## Abstract

The non-linear voltage-dependent hysteresis (Hys_(V)_) of voltage-gated ionic currents can be robustly activated by the isosceles-triangular ramp voltage (V_ramp_) through digital-to-analog conversion. Perturbations on this Hys_(V)_ behavior play a role in regulating membrane excitability in different excitable cells. A variety of small molecules may influence the strength of Hys_(V)_ in different types of ionic currents elicited by long-lasting triangular V_ramp_. Pirfenidone, an anti-fibrotic drug, decreased the magnitude of *I*_h_’s Hys_(V)_ activated by triangular V_ramp_, while dexmedetomidine, an agonist of α_2_-adrenoceptors, effectively suppressed *I*_h_ as well as diminished the Hys_(V)_ strength of *I*_h_. Oxaliplatin, a platinum-based anti-neoplastic drug, was noted to enhance the *I*_h_’s Hys_(V)_ strength, which is thought to be linked to the occurrence of neuropathic pain, while honokiol, a hydroxylated biphenyl compound, decreased *I*_h_’s Hys_(V)_. Cell exposure to lutein, a xanthophyll carotenoid, resulted in a reduction of *I*_h_’s Hys_(V)_ magnitude. Moreover, with cell exposure to UCL-2077, SM-102, isoplumbagin, or plumbagin, the Hys_(V)_ strength of *erg*-mediated K^+^ current activated by triangular V_ramp_ was effectively diminished, whereas the presence of either remdesivir or QO-58 respectively decreased or increased Hys_(V)_ magnitude of M-type K^+^ current. Zingerone, a methoxyphenol, was found to attenuate Hys_(V)_ (with low- and high-threshold loops) of L-type Ca^2+^ current induced by long-lasting triangular V_ramp_. The Hys_(V)_ properties of persistent Na^+^ current (*I*_Na(P)_) evoked by triangular V_ramp_ were characterized by a figure-of-eight (i.e., ∞) configuration with two distinct loops (i.e., low- and high-threshold loops). The presence of either tefluthrin, a pyrethroid insecticide, or *t*-butyl hydroperoxide, an oxidant, enhanced the Hys_(V)_ strength of *I*_Na(P)_. However, further addition of dapagliflozin can reverse their augmenting effects in the Hys_(V)_ magnitude of the current. Furthermore, the addition of esaxerenone, mirogabalin, or dapagliflozin was effective in inhibiting the strength of *I*_Na(P)_. Taken together, the observed perturbations by these small-molecule modulators on Hys_(V)_ strength in different types of ionic currents evoked during triangular V_ramp_ are expected to influence the functional activities (e.g., electrical behaviors) of different excitable cells in vitro or in vivo.

## 1. Introduction

Previous electrophysiological measurements with voltage-clamp maneuvers have used rectangular waveforms with varying durations of command voltages to evoke different types of voltage-gated ionic currents in attempts to evaluate the quasi-steady-state relationship of current versus voltage in specified ionic currents. However, recent investigations have revealed that through efficient data acquisition with digital-to-analog conversion, the voltage-clamp protocol with different waveforms (e.g., triangular ramp voltage (V_ramp_)) can be specifically designed and exploited, and as whole-cell configuration was established, the voltage protocol can be thereafter applied to the tested cells. As a result, the non-linear relationship of current trace versus membrane potential (i.e., voltage-dependent hysteresis (Hys_(V)_) can be activated. Such voltage dependence of different ionic currents can shift to either negative or positive potentials following activation, displaying a behavior analogous to that of ferromagnetic materials [1,2]. Of note, the Hys_(V)_’s phenomenon residing in different types of transmembrane ionic currents has been viewed to be linked to conformational changes in the voltage sensor of the channel specified, and it has been also demonstrated to play an essential role in influencing the electrical behaviors of variable excitable cells [2].

In this review article, we intended to demonstrate that several intriguing small molecules interact with different types of transmembrane ionic currents to alter the behavior of Hys_(V)_. The non-equilibrium Hys_(V)_ properties residing in different types of ionic currents were mostly activated by the upright or inverted isosceles-triangular V_ramp_ through digital-to-analog conversion. The ionic currents involved include hyperpolarization-activated cation current (*I*_h_), *erg*-mediated K^+^ current (*I*_K(erg)_), M-type K^+^ current (*I*_K(M)_), L-type Ca^2+^ current (*I*_Ca,L_), and persistent Na^+^ current (*I*_Na(P)_) (Table 1). The Hys_(V)_ occurrence induced during triangular V_ramp_ is thought to reflect that a mode shift during channel activation may exist since the voltage sensitivity of the gating charge movement relies on the previous state (conformation) of the channel involved [2]. Several small-molecule modulators have been found to regulate the Hys_(V)_ strength occurring in different types of ionic currents (Table 1).

## 2. Hys_(V)_ Behavior Residing in Hyperpolarization-Activated Cation Current (*I*_h_)

### 2.1. Pirfenidone (Esbriet^®^, 5-Methyl-1-Phenylpyridin-2[H-1]-One)

The magnitude of *I*_h_ (or funny current [I_f_]) has been viewed to be a notable determinant of repetitive electrical activities inherently in heart cells and various excitable cells [3,4,5,6,7,8,9]. This type of ionic current is characterized by a mixed inward Na^+^/K^+^ current with a slowly activating property during long-lasting membrane hyperpolarization. Pirfenidone is thought to act by interfering with the production of transforming growth factor-β and tumor necrosis factor-α and it is a new anti-fibrotic drug for idiopathic pulmonary fibrosis [10]. Of note, a recent paper has convincingly demonstrated the ability of pirfenidone to produce a reduction in the Hys_(V)_’s strength of *I*_h_ evoked by long-lasting inverted triangular V_ramp_ [11,12]. In other words, there was a substantial reduction in ∆area of *I*_h_’s Hys_(V)_ loop encircled by the forward and backward limbs of the inverted double V_ramp_. The experimental results thus suggest that cell exposure to pirfenidone can diminish such Hys_(V)_ entailed in the voltage-dependent elicitation of *I*_h_. The inhibitory effect of pirfenidone on *I*_h_ was also accompanied by substantial depression in the magnitude of sag voltage elicited by hyperpolarizing current stimulus as observed under current-clamp potential recordings. However, neither the amplitude of *I*_K(M)_ nor *I*_K(erg)_ was altered by the presence of this compound. Therefore, these results highlight evidence that pirfenidone is capable of perturbing the magnitude, gating kinetics, and Hys_(V)_ properties of *I*_h_, thereby revealing a potential additional impact on the functional activities (e.g., discharge patterns) of different excitable cells.

### 2.2. Dexmedetomidine

Dexmedetomidine, a lipophilic imidazole derivative, is a potent and selective agonist of α_2_-adrenergic receptors [13]. This drug has been disclosed to exert a variety of actions on the human brain such as sedation, anesthetic sparing effects, and analgesia [13,14]. A recent investigation has shown that dexmedetomidine could perturb on the non-equilibrium property of *I*_h_ in response to triangular V_ramp_ found in GH_3_ cells [15]. The presence of this agent was found to diminish such Hys_(V)_ linked to the voltage-dependent elicitation of *I*_h_. However, further application of yohimbine, dexmedetomidine failed to attenuate dexmedetomidine-mediated reduction in the Hys_(V)_’s area of *I*_h_. Yohimbine is an antagonist of α-adrenergic receptors. As such, the inhibition of *I*_h_’s Hys_(V)_ caused by dexmedetomidine is not associated with a mechanism highly linked to its interaction with α_2_-adrenergic receptors, although pituitary cells were previously demonstrated to express those receptors [16]. It has been reported that HCN2, HCN3, or mixed HCN2+HCN3 channels are intrinsically expressed in GH_3_ cells or other types of endocrine or neuroendocrine cells [4,5,17,18]. Because of the importance of *I*_h_ (i.e., HCNx-encoded currents) in contributing to the excitability and automaticity in different excitable cells [3,4,7,17,18], findings from this study could provide additional but important insights into electrophysiological and pharmacological properties of dexmedetomidine or other structurally similar compounds (e.g., medetomidine). Dexmedetomidine that viably and directly targets ionic channels [15,19] is therefore expected to have a significant therapeutic potential. However, whether dexmedetomidine-induced bradycardia or different cardioprotective action [20] is pertinent to its inhibitory effect on the magnitude and Hys_(V)_ of *I*_h_ intrinsically in heart cells warrants further investigations. 

### 2.3. Oxaliplatin

Oxaliplatin (Eloxatin^®^) belongs to a family of platinum-based chemotherapeutic compounds. Despite the fair safety profile,prolong treatment of oxaliplatin could induce severe peripheral neuropathy, affecting sensory and motor nerve fibers [21,22,23,24,25]. In agreement with previous observations [26,27], the *I*_h_ natively existing in GH_3_ cells was demonstrated to undergo either a Hys_(V)_ change, or a mode shift in situations where the voltage sensitivity in gating charge movements of the current depends on the previous state of the channel [7,11,12]. Recent investigations have clearly demonstrated that the presence of oxaliplatin was capable of enlarging such Hys_(V)_’s ∆area involved in the voltage-dependent elicitation of *I*_h_ [11]. Furthermore, subsequent addition of ivabradine, but still in the continued presence of oxaliplatin, could attenuate oxaliplatin-mediated increase in the ∆area of Hys_(V)_ in response to triangular V_ramp_ [11]. Ivabradine has been reported to be an inhibitor of *I*_h_ [18,28,29,30]. Therefore, the oxaliplatin actions occurring in vivo are not exclusively connected to the formation of platinum-DNA adducts. The perturbations by oxaliplatin on Hys_(V)_ change of *I*_h_ is thus another intriguing mechanism, through which it or other structurally related compounds can interfere with cell behaviors, particularly in electrically excitable cells [21,22,23,25].

### 2.4. Honokiol

Honokiol, a hydroxylated biphenyl compound obtained from *Magnolia officinalis* and from other species of the family Magnoliaceae, has been used in traditional Asian medicine [31]. In a recent study, the authors exploited a long-lasting triangular V_ramp_ for the measurement of the Hys_(V)_ properties in *I*_h_. In this study, as whole-cell configuration was achieved, it is clear that the trajectory of *I*_h_ in response to the upsloping (i.e., depolarizing from −150 to x40 mV) and downsloping (hyperpolarizing from −40 to −150 mV) V_ramp_ as a function of time was distinguishable between these two limbs of triangular V_ramp_ [11,12,26,32]. Importantly, honokiol was capable of diminishing Hys_(V)_’s strength involved in the voltage-dependent activation of *I*_h_. Moreover, with the continued presence of honokiol, the further application oxaliplatin could attenuate honokiol-mediated decrease of the ∆area of the Hys_(V)_ in response to triangular V_ramp_. Oxaliplatin was previously reported to enhance the Hys_(V)_ strength of *I*_h_ [11,12,30]. However, although the voltage ranges in which *I*_h_ activation occurs, either in control conditions or after the honokiol treatment, appear to fall outside of the values of the membrane in a neuron, it needs to be noted that a small fraction of *I*_h_ is tonically activated at rest [33]. Moreover, since the macroscopic *I*_h_ in GH_3_ cells could be a mixture of several channel currents (i.e., HCNx-encoded current), whether honokiol can affect either *I*_h_ existing in a variety of cells or different types of *I*_h_ remains to be rigorously evaluated. The extent to which the honokiol-induced inhibition of *I*_h_ along with its perturbations on Hys_(V)_ contributes to anti-inflammatory or antinociceptive action [25,34,35] is yet to be explored. 

### 2.5. Lutein (Xanthophyll, β,ε-Carotene-3,3′-Diol or 3,3′-Di-Hydroxy-β,α-Carotene)

**Figure 1:**
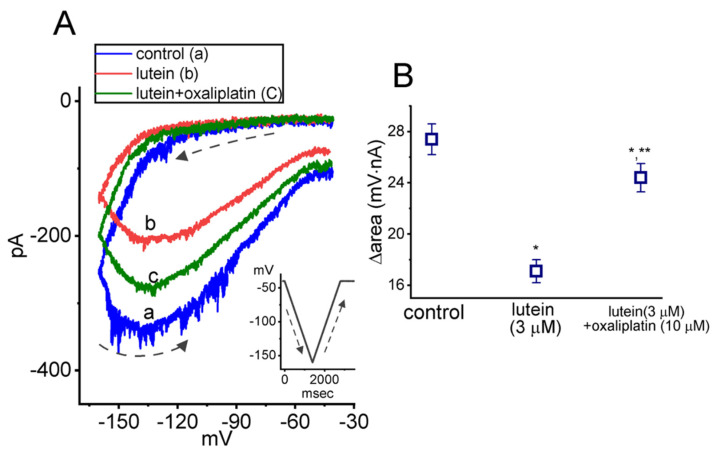
Effect of lutein and lutein plus oxaliplatin on Hys_(V)_ of *I*_h_ measured from pituitary GH_3_ cells. The experiments were conducted in cells bathed in Ca^2+^-free Tyrode’s solution, and the recording pipette was filled up with K^+^-containing solution. The tested cell was held at −40 mV and the inverted isosceles-triangular V_ramp_ from −40 to −150 mV with a duration of 3.2 s (or ramp speed of ±69 mV/s) was thereafter applied to evoke *I*_h_’s Hys_(V)_. (**A**) Representative Hys_(V)_’s traces of *I*_h_ (i.e., the relation of forward [descending] or backward [ascending] current versus membrane potential). a: control (blue color); b: 3 μM lutein (red color); and c: 3 μM lutein plus 10 μM oxaliplatin (green color). Inset indicates the voltage protocol imposed. The black dashed arrows underneath the current traces in the control period (i.e., neither lutein nor oxaliplatin was present) indicate *I*_h_ trajectory in an anti-clockwise direction when time passes during the inverted triangular V_ramp_. (**B**) Summary graph disclosing effects of lutein (3 μM) and lutein (3 μM) plus oxaliplatin (10 μM) on the ∆area of *I*_h_’s Hys_(V)_ (i.e., the curves encircled by *I*_h_’s Hys_(V)_ activated during the descending and ascending limb of the triangular V_ramp_). * Significantly different from control (*p* < 0.05) and ** significantly different from lutein (3 μM) alone group (*p* < 0.05).

The Hys_(V)_ properties of *I*_h_ activated by triangular Vramp were known to perturb the electrical behaviors of various excitable cells [6,26,30]. Voltage-sensing domain relaxation in the channel proteins (e.g., HCNx channels) has been noticed to involve in such Hys_(V)_ behavior [26,36]. Alternatively, the observed “inertia” in the responsiveness of HCNx channels can be driven by changes in their electrical sensitivity, which is presumably allowed to resemble that occurring in ferromagnetic materials displaying Hys_(V)_ behaviors [1,2]. Of notice, the *I*_h_ intrinsically residing in GH_3_ cells underwent a non-equilibrium property of instantaneous *I*_h_. That is, there appears to be an anti-clockwise Hys_(V)_ loop responding to the isosceles-triangular Vramp as demonstrated in Figure 1A. Such perturbations have been viewed to be dynamically linked to a mode shift in situations where the voltage sensitivity of gating charge movements (i.e., voltage-sensing domain relaxation) depends on the previous state (or conformation) of the channel (e.g., HCNx channel). Of additional interest, GH_3_-cell exposure to lutein resulted in a significant reduction in Hys_(V)_ strength of *I*_h_ evoked by long-lasting inverted triangular Vramp (Figure 1A,B). Upon continued exposure to lutein (3 μM), the subsequent addition of oxaliplatin (10 μM) was able to attenuate lutein-mediated decrease in the ∆area of *I*_h_’s Hys_(V)_ observed in these cells. Oxaliplatin, a platinum-based anti-neoplastic agent, has been demonstrated to be an activator of *I*_h_ [11,23]. The presence of lutein effectively suppressed the magnitude of *I*_h_ in pituitary GH_3_ cells with an IC_50_ value of 4.1 μM. Under current-clamp potential recordings, the sag potential evoked by long-lasting hyperpolarizing current stimulus also became reduced during cell exposure to this compound [37]. Lutein is one of the few xanthophyll carotenoids which exist not only in vegetables and fruits, but is also enriched in the macular of the human retina [38]. 

Moreover, based on the docking prediction, it is likely that the interaction of the lutein molecules with HCN channels could be located at the cytosolic side of the membrane [37]. Lutein may thus bind to the HCNx channels and interfere with channel gating to alter the magnitude, gating and Hys_(V)_ of *I*_h_. Findings from these recent reports tempt us to propose that the *I*_h_ present in different cell types could thus be unidentified, but the lutein molecules can act through distinctive targets to affect the functional activities of the cells involved. Nonetheless, lutein-mediated decrease in the Hys_(V)_’s area is thought to be strongly linked to the voltage-dependent elicitation of HCN channel [7,27,36]. However, either whether the lutein molecules can interact mainly with the voltage-sensing domains of HCNx channels [7] to alter Hys_(V)_ strength of the channel, or how lutein-mediated changes in the Hys_(V)_’s strength influence the functional activities (e.g., electrical behaviors) in variable excitable cells, still remains to be explored. 

Additionally, HCN channels have been previously demonstrated to be linked to phototransduction in photosensitive retinal ganglion cells [39]. Its activity was found either to alter the electroretinographic ON and OFF responses or to delay photoreceptor degeneration [40]. To what extent lutein-mediated changes in Hys_(V)_ behavior of *I*_h_ is associated with its action on age-related diseases (e.g., macular degeneration) [41] still needs to be further determined.

## 3. Hys_(V)_ Behavior Residing in *Erg*-Mediated K^+^ Current (*I*_K(erg)_)

### 3.1. UCL-2077 (3-(Triphenylmethylaminomethyl)pyridine))

The *I*_K(erg)_ encoded by three different subfamilies of the gene KCNH is known to give rise to the pore-forming α-subunit of erg-mediated K^+^ (i.e., K_erg_ or K_V_11) channels. These macroscopic currents are regarded to constitute the cloned counterpart of the rapidly activating delayed-rectifying K^+^ currents in heart cells, where the KCNH2 gene encodes the pore-forming α-subunit of the K_V_11.1 channels, commonly identified as hERG [42,43]. These currents inherently existing in neurons or in different types of electrically excitable cells, such as endocrine or neuroendocrine cells, can highly influence the maintenance of the resting potential as well as the increase in subthreshold excitability [44,45]. In GH_3_ cells bathed in Ca^2+^-free high-K^+^ solution, as whole-cell configuration in the patch-clamp current recordings was established, the examined cell was hyperpolarized from −10 to long-lasting hyperpolarization (e.g., 1 s) and the deactivating *I*_K(erg)_ with a slowly decaying time course can be robustly elicited [30,44,46]. Moreover, the Hys_(V)_ properties present in *I*_K(erg)_ have been proposed to play a role in influencing the electrical behavior of excitable cells. In an earlier study, consistent with previous observations in HCN channels [26,27,36], K_erg_ channels inherently existing in GH_3_ cells were noticed to undergo either a Hys_(V)_ in their voltage dependence or a mode-shift, in which the voltage sensitivity of gating charge movements depends on the previous state [47,48]. The *I*_K(erg)_’s Hys_(V)_ reflects that a mode shift during channel activation may exist because the voltage sensitivity of the gating charge movement depends on the previous state (conformation) of K_erg_ channels. Under such a scenario, when the membrane potential becomes negative (i.e., the downward limb of the inverted triangular V_ramp_), the voltage dependence of K_erg_ channel may shift the mode of Hys_(V)_ to one which occurs at more negative potentials, thereby leading to an increase in membrane repolarization. However, as the membrane potential is depolarized (i.e., during initiation of action potentials or upward end of the triangular V_ramp_), the voltage-dependence of *I*_K(erg)_ activation would quickly switch to less depolarized voltages with a smaller current magnitude, thereby having the tendency to increase membrane excitability [47]. The experimental results also revealed that the presence of UCL-2077 was able to decrease Hys_(V)_’s strength of *I*_h_ elicitation by triangular V_ramp_ [48]. Although the underlying mechanism of neuronal slow after-hyperpolarization is currently unclear, previous studies demonstrated that the ability of UCL-2077 in slow modification after-hyperpolarization [49] could be, partly if not entirely, attributed to its modifications on the magnitude, gating kinetics, and Hys_(V)_ behavior of V_ramp_-induced *I*_K(erg)_.

### 3.2. SM-102 (1-Octylnonyl 8-[(2-Hydroxyethyl)[6-oxo-6(Undecyloxy)hexyl]amino]-Octanoate)

SM-102 is a synthetic and ionizable amino lipid that has been widely used in combination with other lipids in the formation of lipid nanoparticles [50,51,52]. Formulations containing SM-102 have been noticeably used in the development of lipid nanoparticles for the delivery of mRNA-based vaccines. For example, SM-102 is known to be one of the ingredients in the Moderna^TM^ COVID-19 vaccine [52]. Recent investigations have also disclosed that the strength of Hys_(V)_ of *I*_K(erg)_ elicited by the upright isosceles-triangular V_ramp_ was profoundly decreased as cells were exposed to SM-102 or TurboFectin^TM^ [53]. TurboFectin^TM^ is a proprietary mixture of a broad-spectrum protein/polyamine with histones and lipids, which is known to be a transfection reagent. Moreover, with continued exposure to SM-102 or TurboFectin^TM^, further application of PD118057 was able to attenuate the inhibition by these two agents on *I*_K(erg)_’s strength activated during the triangular V_ramp_. PD118057 was previously reported to be an activator of *I*_K(erg)_ [54]. The magnitude of inwardly rectifier K^+^ currents inherently in BV2 microglial cells was also subjected to be inhibited by SM-102. In sum, SM-102 concentration-dependently suppressed *I*_K(erg)_ magnitude in endocrine cells (e.g., GH_3_ or MA-10 cells) along with the decrease of Hys_(V)_’s strength of the current [53]. These above actions are thus anticipated to contribute to their functional effects on different cell types, presumably similarly affected in vitro or in vivo.

### 3.3. Isoplumbagin (5-Hydroxy-3-Methyl-1,4-Naphthoquinone) and Plumbagin (5-Hydroxy-2-Methyl-1,4-Naphthoquinone)

Isoplumbagin is a naturally occurring quinone from *Lawsonia inermis* or *Plumbago europaea*, while plumbagin, another hystodyl-1,4-naphthoquinone, is an alkaloid obtained from the roots of the plants of the Plumbago genus. Isoplumbagin and plumbagin have recently been demonstrated to exert anti-neoplastic activity against an array of cancers [55,56]. Earlier studies have revealed that the *I*_K(erg)_ residing in GH_3_ cells did undergo Hys_(V)_ behavior activated during the inverted isosceles-triangular V_ramp_, reflecting that the K_erg_ channels in these cells display a clear Hys_(V)_ in the voltage dependence, which is closely linked to the voltage sensor domain inherently in the channel [48,53,57]. Moreover, upon cell exposure to isoplumbagin or plumbagin, the ∆area (i.e., the area encircled by the Hys_(V)_ curves elicited by the descending and ascending direction) of *I*_K(erg)_’s Hys_(V)_ during the inverted triangular V_ramp_ was markedly reduced [57]. Isoplumbagin was also demonstrated to suppress *I*_K(erg)_ magnitude in MA-10 Leydig tumor cells [57]. Therefore, the inhibition by isoplumbagin or plumbagin of *I*_K(erg)_’s magnitude and Hys_(V)_’s strength would be expected to have an important impact on the discharge patterns of actions potentials occurring in excitable cells. Docking results have additionally shown that there appears to be a predicted interaction (i.e., the formation of hydrogen bond and hydrophobic contacts) between the isoplumbagin or plumbagin molecule and hERG channel [57]. In this regard, isoplumbagin, plumbagin, or other structurally similar compounds [58] could be intriguing compounds useful for characterizing the K_erg_ channels. Moreover, it remains to be studied whether this ionic mechanism of their actions on *I*_K(erg)_ described presently can be closely linked to their actions on either functional activities or aberrant growth of different neoplastic cells [59,60]. 

## 4. Hys_(V)_ Behavior Residing in M-Type K^+^ Current (*I*_K(M)_)

### 4.1. Remdesivir (Development Code: GS-5734)

It has been shown that the KCNQ2, KCNQ3, or KCNQ5 encodes the core subunit of K_V_7.2, K_V_7.3, or K_V_7.5 channels. The enhanced activity of this family of K^+^ channels (KCNQx, K_V_7x, or K_M_ [M-type K^+^] channels) can generate macroscopic M-type K^+^ current (*I*_K(M)_) [30,61,62,63]. Once evoked during membrane depolarization, the currents have been disclosed to exhibit a slowly activating and deactivating property as well as to affect the bursting patterns in different types of neurons, endocrine and neuroendocrine cells [30,63,64,65]. Remdesivir, a broad-spectrum antiviral agent, is recognized as a mono-phosphoramidate prodrug of an adenosine analog that metabolizes into its active form GS-441524 which is a C-adenosine nucleoside analog [66]. This compound, a nucleotide-analog inhibitor of RNA-dependent RNA polymerase, is thought to be highly active against coronaviruses (CoVs), including MERS-Cov and SARS CoV-2 [67]. The recent investigations have disclosed that remdesivir could suppressed the magnitude of *I*_K(M)_ in pituitary GH_3_ cells [68]. Moreover, the magnitude of *I*_K(M)_’s Hys_(V)_ elicited by long-lasting triangular V_ramp_ was diminished by adding remdesivir. In Jurkat T-lymphocytes, remdesivir could effectively decrease the amplitude of delayed-rectifier K^+^ current concomitantly with the raised rate of current inactivation evoked by step depolarization. As such, in terms of the remdesivir molecule itself, there seems to be an unintentional activity of the prodrug on *I*_K(M)_. The perturbing effects of remdesivir on membrane ionic currents were noted to be rapid in onset, and they should be upstream of its actions occurring inside the cytosol or nucleus. Its inhibition of *I*_K(M)_’s Hys_(V)_ emerging in a non-genomic fashion might provide additional but important mechanisms through which in vivo cellular functions are perturbed.

### 4.2. QO-58 (5-(2,6-Dichloro-5-Fluoropyridin-3-yl)-3-Phenyl-2-(Trifluoromethyl)-1H-Pyrazolol[1,5-a]pyrimidin-7-One)

The Hys_(V)_ behavior of ionic currents has been recently noticed to exert important impacts on electrical behaviors of action potential firing [26,27,62,63]. The *I*_K(M)_ intrinsically residing in GH_3_ cells was robustly observed to undergo V_ramp_-induced Hys_(V)_ [65], suggesting that the voltage sensitivity of gating charge movements relies on the previous state (or conformation) of the M-type K^+^ (K_M_) channel. Alternatively, as the membrane potential of the cell becomes depolarized (i.e., during initiation of an action potential or the upsloping limb of the triangular V_ramp_), the voltage dependence of *I*_K(M)_ activation would switch to less depolarized voltage with a small current magnitude, thereby causing the depression of membrane excitability. However, as the membrane potential becomes negative (i.e., downward V_ramp_), the voltage dependence of K_M_ channels may shift the mode of Hys_(V)_ to one which occurs at more negative potentials, thereby resulting in an increase in membrane repolarization. Moreover, upon triangular V_ramp_ with varying durations, QO-58 increased the Hys_(V)_’s strength of *I*_K(M)_ [65]. QO-58 has been demonstrated previously to be an opener of KCNQx (K_V_7x) channels [65,69,70]. In this regard, the experimental observations led to the notion that there would be a perturbing stimulatory effect of QO-58 on such non-equilibrium property (i.e., non-linear Hys_(V)_ behavior) in K_M_ (or K_V_7) channels in electrically excitable cells. However, how QO-58-induced modifications on *I*_K(M)_’s Hys_(V)_ are linked to the behavior of these cells occurring in vivo remains to be further resolved.

## 5. Hys_(V)_ Behavior Residing in L-Type Ca^2+^ Current (*I*_Ca,L_)

### Zingerone (Ginerone, Vanillylacetone)

Zingerone is a nontoxic methoxyphenol isolated from the rhizome of ginger (*Zingiber officinale* Roscoe), and it has been used as a flavor additive in spiced oils and in perfumery to introduce exotic aromas. It is widely viewed to have potential anti-inflammatory, anti-diabetic, antilipolytic, antidiarrheal, antispasmodic, and anti-tumor properties [71]. In a recent study, pituitary GH_3_ cells were kept in normal Tyrode’s solution containing 1.8 mM CaCl_2_, and when an abrupt double V_ramp_ was applied to the tested cell, there appeared a Hys_(V)_ loop with a figure-of-eight pattern of L-type Ca^2+^ current (*I*_Ca,L_) [72]. The Hys_(V)_ properties of *I*_Ca,L_ are noted to be distinguishable from those described above in either *I*_h_, *I*_K(erg)_ or *I*_K(M)_ evoked by triangular V_ramp_. In other words, the trajectory of the instantaneous current induced by V_ramp_ revealed two loops, namely, a high-threshold anticlockwise and a low-threshold clockwise loop, during Hys_(V)_ elicitation. However, as extracellular Ca^2+^ was replaced with Ba^2+^ ions, the low-threshold current at the downsloping phase of triangular V_ramp_ diminished, whereas the high-threshold current at the upsloping end of V_ramp_ became increased. The formation of a low-threshold clockwise loop was thought to be attributed either to the magnitude of the Ca^2+^-activated nonselective cationic currents or the late component of *I*_Ca,L_ [73,74]. Consequently, the replacement of Ca^2+^ ions with Ba^2+^ ions increased the amplitude of *I*_Ca,L_ (i.e., barium inward current, *I*_Ba_) activated by rectangular depolarization from −50 to +10 mV, in combination with a conceivable slowing in inactivation process of the current. However, the Hys_(V)_ of the current activated by the double V_ramp_ was reduced during the high-amplitude loop of V_ramp_, as well as it was concurrently increased at the low-amplitude loop [72]. Of additional note, as cells were exposed to zingerone, the area (encircled by *I*_Ca,L_’s Hys_(V)_) of both high- and low-threshold loop of *I*_Ca,L_ activated by the V_ramp_ were markedly reduced. Whether zingerone-mediated inhibition of *I*_Ca,L_ accompanied by the decreased Hys_(V)_ strength of the current can be responsible for its potential to attenuate seizure activity [75,76], remains to be further evaluated.

## 6. Hys_(V)_ Behavior Residing in Persistent Na^+^ Current (*I*_Na(P)_)

### 6.1. Esaxerenone (Minnebro^®^)

Esaxerenone, known to be a newly oral, non-steroidal selective blocker on the activity of mineralocorticoid receptor, has been growingly used for the management of various pathological disorders, such as primary aldosteronism, refractory hypertension, chronic kidney disease, diabetic nephropathy, and heart failure [77,78,79]. In a recent investigation, the addition of esaxerenone to pituitary GH_3_ cells suppressed the transient (*I*_Na(T)_) and late component (*I*_Na(L)_) of *I*_Na_ with effective IC_50_ of 13.2 and 3.2 μM, respectively [80]. Furthermore, the non-linear Hys_(V)_ of V_ramp_-induced *I*_Na(P)_ in the control period (i.e., neither tefluthrin nor esaxerenone was present) and during cell exposure to tefluthrin or tefluthrin plus esaxerenone was observed by the upright isosceles-triangular V_ramp_ with varying durations. In particular, when cells were exposed to 10 μM tefluthrin, the peak *I*_Na(P)_ amplitude activated at the forward (upsloping) limb of the triangular V_ramp_ was noted to increase, particularly at the level of −30 mV, whereas the *I*_Na(P)_ amplitude at the backward (downsloping) end at −80 mV arose. In this regard, distinguishable from Hys_(V)_ configuration present in *I*_h_, *I*_K(erg)_ and *I*_K(M)_ elaborated above, the instantaneous figure-of-eight (i.e., infinity-shaped: ∞) configuration residing in the *I*_h_’s Hys_(V)_ loop during upright triangular V_ramp_ appeared. These results indicate that, as the time goes by during activation, there is a counterclockwise direction in the high-threshold loop (i.e., the relationship of current amplitude as a function of membrane potential), followed by a clockwise direction in the low-threshold loop. Consequently, in the presence of 10 μM tefluthrin, the figure-of-eight configuration in the Hys_(V)_ loop elicited by the triangular V_ramp_ was demonstrated and further enhanced. Tefluthrin, a type-I pyrethroid insecticide, has been previously demonstrated to be an activator of *I*_Na_ accompanied by the slowed inactivation of the current [81,82,83]. In other words, there appeared to be the two distinct types of *I*_Na(P)_, i.e., low-threshold (i.e., activating at a voltage range near the resting potential of the cell) and high-threshold loop (i.e., activating at a voltage range near the maximal *I*_Na_ achieved), clearly observed ruing cell exposure to tefluthrin. Of note, the low-threshold *I*_Na(P)_ was identified to be activated (at the voltage range near the resting potential) upon the downsloping end of the triangular ramp pulse. However, the high-threshold *I*_Na(P)_ (at the voltage range where peak *I*_Na(T)_ was maximally activated) was by the upsloping end of such V_ramp_. As the ramp speed decreased with a lowering in peak V_ramp_, the area of such Hys_(V)_ became progressively reduced. Therefore, finding from these results revealed that the *I*_Na(P)_ elicited by triangular V_ramp_ was observed to undergo Hys_(V)_ changes in the voltage-dependence found in GH_3_ cells [80].

In an earlier study, as GH_3_ cells were exposed to tefluthrin, the voltage-dependent movement of S4 segment residing in Na_V_ channels could be perturbed; as a result, the coupling of the pore domain to the voltage-sensor domain was enhanced [83]. Such unique type of Hys_(V)_ behavior inherently in Na_V_ channels would potentially play substantial role either in influencing electrical behaviors, Na^+^ overload due to an excessive Na^+^ influx, or in hormonal secretion in various types of excitable cells during exposure to pyrethroid insecticides (e.g., tefluthrin or other structurally similar synthetic pyrethroids [e.g., deltamethrin, metofluthrin, and permethrin]). Additionally, the subsequent addition of esaxerenone, but still during continued exposure to tefluthrin, was noted to result in a marked attenuation of Hys_(V)_ strength responding to triangular V_ramp_ [80]. The results presented herein are interesting, and they hence led us to propose that, in concert with its antagonistic action of mineralocorticoid receptor, the exposure to esaxerenone may directly modify the magnitude, gating properties, and Hys_(V)_ strength of *I*_Na_ present in different excitable cells. It also needs to be mentioned that the activity of Na_V_ channels has been found to be functionally expressed in various types of vascular smooth muscles [84,85]. Therefore, it is worth pursuing to a further extent as to which esaxerenone-induced antihypertensive action [78,79] is associated with its additional inhibitory action on *I*_Na_ (i.e., Na_V_1.7-encoded current) inherently in vascular smooth myocytes.

**Figure 2:**
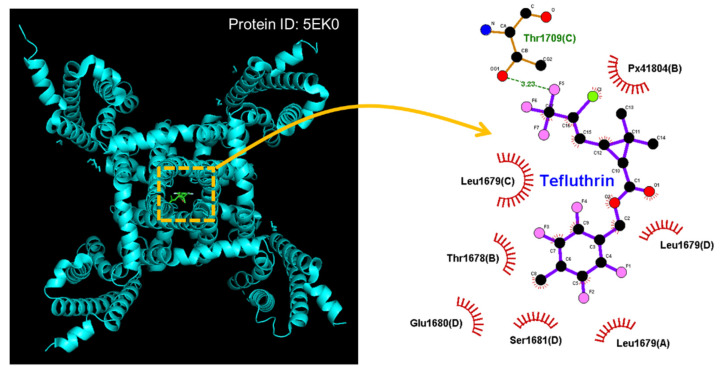
Docking results of the hNa_V_1.7 channel and the tefluthrin molecule. The protein structure of hNa_V_1.7 was acquired from RCB PDB (ID: 5EK0), whereas the chemical structure of tefluthrin was from PubChem (compound CID: 5281874 [3D conformer]). The structure of the hNa_V_1.7 channel was docked by the tefluthrin molecule in PyRx software (http://pyrx.sourceforge.io/) (accessed on 26 July 2022). Diagram of the interaction between the hNa_V_1.7 channel and the tefluthrin molecule generated by LigPlot^+^ (http://www.ebi.ac.uk/thornton-srv/software/LIGPLOT/) (accessed on 26 July 2022). Note that the red arcs on which spokes faced radiating toward the ligand (i.e., tefluthrin) represent hydrophobic interactions, while green dotted line residing in amino-acid residue (i.e., Thr1708(C)) is the formation of a hydrogen bond.

In this study, we also explored how the protein of the hNa_V_1.7 channel could be optimally docked with the tefluthrin molecule by using PyRx software. The protein structure of hNa_V_1.7 was obtained from RCB PDB (ID: 5EK0) [86]. The predicted docking sites of the tefluthrin molecule with which the amino acid residues can interact are presented in Figure 2. It is thus important to note that the tefluthrin molecule may form hydrophobic contacts with certain amino-acid residues, including Thr1678(B), Leu1679(A), Leu1679(C), Leu1679(D), Glu1680(D), Ser1681(D), and Px41804(B) (what is this?). The atom in the tefluthrin molecule has a hydrogen bond with residue Thr1709(C) at a distance of 3.23 Å. On the basis of the Na_V_1.7 [Antrozous pallidus] protein sequence (GenBank: ASY-04966.1, https://www.ncbi.nlm.nih.gov/protein/ASY04966.1?report=gpwithparts&log$=seqview, accessed on 21 August 2022), the inactivation gate of the channel is found to be located at the residue positions ranging between 1459 and 1462, which are adjacent to the docking sites of the tefluthrin molecule. These docking results therefore tempted us to propose that the tefluthrin molecule can dock to the transmembrane segment (position: 1665–1683) of hNa_V_1.7 channel (PDB: 5EK0) with a binding affinity of −7.5 kcal/mol, thereby potentially influencing the magnitude, gating kinetics, and Hys_(V)_ strength of *I*_Na._

### 6.2. Mirogabalin

Mirogabalin (Tarlige^®^) is an orally administered gabapentinoid, and it was thought to be a selective ligand for the α_2_δ-1 subunit of voltage-gated Ca^2+^ channels [87]. More notable than the issue concerning the magnitude of mirogabalin-induced reduction in *I*_Na_, is the current observation of the non-linear Hys_(V)_ of *I*_Na(P)_ elicited by using the upright isosceles-triangular V_ramp_ in pituitary GH_3_ lactotrophs [88]. The presence of mirogabalin in GH_3_ cells caused a concentration-dependent inhibition of *I*_Na(T)_ and *I*_Na(L)_ amplitude with the estimated IC_50_ value of 19.5 and 7.3 μM, respectively [88]. Moreover, during cell exposure to mirogabalin, the peak *I*_Na(P)_ activated by the ascending (upsloping) limb of the triangular V_ramp_ became decreased, particularly at the level of −10 mV, while the *I*_Na(P)_ amplitude at the descending (downsloping) phase was also concurrently reduced at the level of −80 mV. As a result, there turned out to be two distinct types of Hys_(V)_ loop; that is, a high-threshold loop with a peak at −10 mV (i.e., activating at a voltage range near the maximal amplitude of transient Na^+^ current (*I*_Na(T)_ evoked by brief step depolarization), and a low-threshold loop with a peak at −80 mV (i.e., activating at a voltage near the resting membrane potential). The application of mirogabalin was able to attenuate the Hys_(V)_ strength of *I*_Na(P)_ effectively [88]. Under this scenario, the observations reveal that the triangular V_ramp_-induced *I*_Na(P)_ undergoes striking Hys_(V)_ behavior with the voltage dependence, and that such Hys_(V)_ loops responding to triangular V_ramp_ are subjected to attenuation by adding mirogabalin. The Hys_(V)_ behavior of *I*_Na(P)_ existing in endocrine or neuroendocrine cells in vivo could be strongly linked to the magnitude of Na^+^ background currents, as reported previously [17,27,82,89,90,91,92,93,94,95]. Alternatively, genetic defects (i.e., gain-of-function) in Na_V_ channel inactivation that led to small, sustained *I*_Na(P)_, are recognized to have devastating consequences, including neuropathic pain and convulsant activity [89,90,94,96,97,98].

### 6.3. Dapagliflozin (Foxiga^®^)

**Figure 3:**
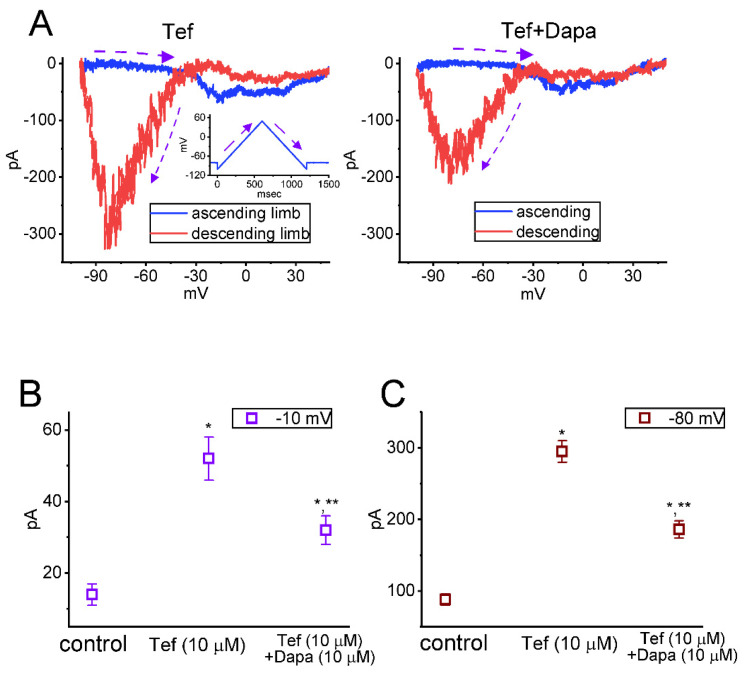
Effect of tefluthrin (Tef) and Tef plus dapagliflozin (Dapa) on Hys_(V)_ loop of *I*_Na(P)_ in pituitary GH_3_ lactotrophs. In these experiments, we placed cells in the Ca^2+^-free Tyrode’s solution containing 10 mM tetraethylammonium chloride and 0.5 mM CdCl_2_, and the recording electrode was filled with Cs^+^-enriched solution. (**A**) Representative current traces are activated by the upright isosceles-triangular V_ramp_ for a duration of 1.2 sec, or with a ramp speed of 125 mV/s (as indicated in inset of left part). The blue color in the left and right part represents the current trace activated by the ascending (upsloping) limb of the V_ramp_, the red color indicates the trace by the V_ramp_’s descending (downsloping) end, and the purple dashed arrow adjacent to potential or current trace demonstrates the direction of the potential or current over which time goes during the elicitation of the long-lasting triangular V_ramp_. Of note, there is a unique Hys_(V)_ loop (i.e., the figure of eight configuration) evoked by the isosceles-triangular V_ramp_ obtained in the presence of tefluthrin (Tef, 10 μM) or tefluthrin plus dapagliflozin (Dapa, 10 μM). In (**B**,**C**), summary graphs, respectively, depict effects of Tef or Tef plus Dapa on the amplitude of *I*_Na(P)_ activated by the upsloping (at −10 mV) and downsloping (at −80 mV) limbs of the triangular V_ramp_ (mean ± SEM; *n* = 8 for each point). * Significantly different from control (*p* < 0.05), and ** significantly from Tef (10 μM) alone group (*p* < 0.05). Of note, the magnitude appearing in (**B**,**C**) is indicated as the absolute value of current amplitude.

Dapagliflozin is viewed to be a selective inhibitor of Na^+^-dependent glucose co-transporter (SGLT) that can block glucose transport which is highly selective for SGLT2 over SGLT1 [99,100,101]. However, an earlier report has shown the capability of empagliflozin, another structurally similar compound, in blocking cardiac late Na^+^ currents [102]. Of additional notice, the recent observations at our laboratory found that further application of dapagliflozin (10 μM) in the presence of tefluthrin (10 μM) could effectively and directly attenuate dapagliflozin-enhanced strength of *I*_Na(P)_’s Hys_(V)_ observed in GH_3_ cells (Figure 3). Consistent with previous studies [102], dapagliflozin is effective at suppressing *I*_Na_ as well as at decreasing the strength of *I*_Na(P)_’s Hys_(V)_ in response to the upright isosceles-triangular V_ramp_.

**Figure 4:**
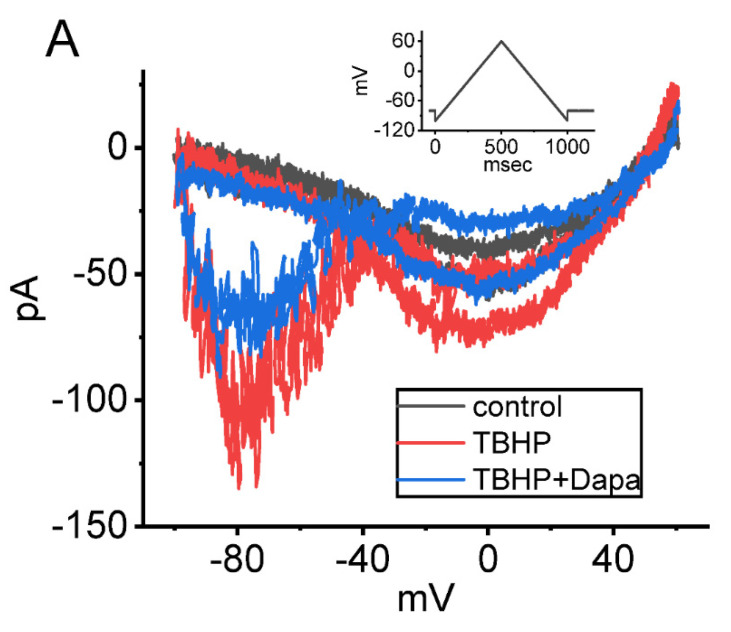

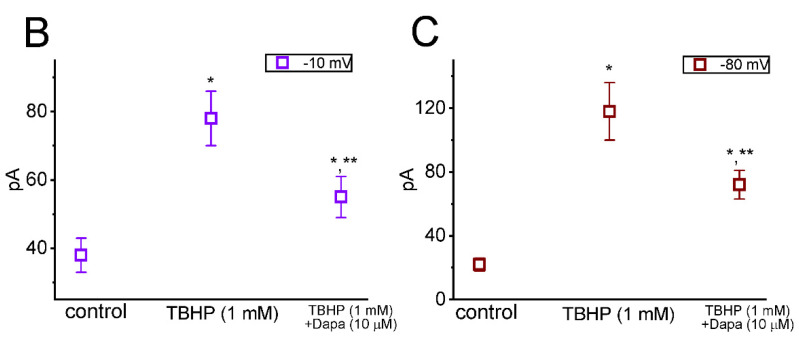
Effect of t-butyl hydroperoxide (TBHP) and TBHP plus dapagliflozin (Dapa) on Hys_(V)_ loop of *I*_Na(P)_ in pituitary GH_3_ lactotrophs. (**A**) Representative current traces activated by the triangular V_ramp_ for a duration of 1 s (or ramp speed of 320 mV/s) (as indicated in inset). Current trace shown in black color is control (i.e., neither TBHP nor Dapa), while that in red or blue color was respectively obtained in the presence of 1 mM TBHP, or 1 mM TBHP plus 10 μM Dapa. Two red and blue traces indicate current trajectories, respectively, activated by the upsloping and downsloping end of the V_ramp_. In (**B**,**C**), summary graphs, respectively, demonstrate effects of TBHP or TBHP plus Dapa on the amplitude of *I*_Na(P)_ at the upsloping (−10 mV) and downsloping (−80 mV) ends of triangular V_ramp_ (mean ± SEM; *n* = 7 for each point). * Significantly different from control (*p* < 0.05), and ** significantly different from TBHP (1 mM) alone group (*p* < 0.05).

The effect of t-butyl hydroperoxide, a hydrophilic oxidant, on Hys_(V)_ of *I*_Na(P)_ was also further examined. As demonstrated in Figure 4, upon cell exposure to 1 mM t-butyl hydroperoxide, the Hys_(V)_’s strength (at the level of −10 and −80 mV) of *I*_Na(P)_ responding to triangular V_ramp_. Furthermore, during the continued presence of 1 mM t-butyl hydroperoxide, further application of dapagliflozin (10 μM) was noticed to reverse t-butyl hydroperoxide-mediated increase of Hys_(V)_’s strength. The results therefore reflect that, consistent with previous investigations [102], the challenge of GH_3_ cells to t-butyl hydroperoxide increased Hys_(V)_ magnitude of V_ramp_-induced *I*_Na(P)_ and the subsequent addition of dapagliflozin counteracted its increase of Hys_(V)_ strength.

It has been demonstrated that the different Na_V_ subtypes (isoforms) can combine to constitute macroscopic *I*_Na_ residing in varying types of excitable cells [103,104]. Na_V_1.1, Na_V_1.2, Na_V_1.3, and Na_V_1.6 channels were previously reported to be expressed in GH_3_ cells [17]. As such, distinguishable to some extent from previous reports demonstrating the ability of empagliflozin, another SGLT2 inhibitor, in inhibiting the late component of cardiac-specific Na^+^ current [102], it seems unlikely that dapagliflozin-induced inhibition of *I*_Na_ inherently in native cells is isoform-specific. Nonetheless, the present results strongly reflect that inhibitory effect of dapagliflozin or other structurally similar compounds (e.g., canagliflozin and empagliflozin) on *I*_Na_, particularly *I*_Na(P)_, which may occur within the clinically therapeutic range, would be another obligate ionic mechanism through which they could converge to perturb the functional activities (e.g., electrical behaviors, Na^+^ influx, and glucose uptake) in different excitable cells.

## 7. Conclusions

As described above and in published studies, the experimental observations have revealed that several voltage-gated ion channels were found to undergo non-linear Hys_(V)_ behavior elicited during triangular V_ramp_. A variety of small molecules (Table 1) known to modify the magnitude and gating of ionic currents (i.e., *I*_h_, *I*_K(erg)_, *I*_K(M)_, *I*_Ca,L_, and *I*_Na(P)_) may pertinently perturb the Hys_(V)_ behavior of the currents. The modifications of Hys_(V)_ exerted by these small-molecule modulators are capable of potentially affecting the functional activities of different excitable cells, presuming that the in-vivo findings occurred.

## Figures and Tables

**Table 1:** Summary in the perturbations of the known small-molecule modulators on voltage-dependent hysteresis (Hys_(V)_) behavior occurring in different types of ionic currents present in excitable cells (e.g., pituitary GH_3_ lactotrophs).

Associated Ionic Currents	Small Molecules
hyperpolarization-activated cation current (*I*_h_)	pirfenidone, oxaliplatin, lutein, dexmedetomidine, honokiol
*erg*-mediated K^+^ current (*I*_K(erg)_)	UCL-2077, SM-102, isoplumbagin, plumbagin
M-type K^+^ current (*I*_K(M)_)	remdesivir, QO-58
L-type Ca^2+^ current (*I*_Ca,L_)	zingerone
persistent Na^+^ current (*I*_Na(P)_)	esaxerenone, tefluthrin, *t*-butyl hydroperoxide, mirogabalin, and dapagliflozin

## Data Availability

The original data is available upon reasonable request to the corresponding author.

## Abbreviations

*erg*	*ether-**à-go-go*-related gene
Hys_(V)_	voltage-dependent hysteresis
HCN channel	hyperpolarization-activated cyclic nucleotide-gated channel
*I*_Ca,L_	L-type Ca^2+^ current
*I*_h_	hyperpolarization-activated cation current
*I*_K(erg)_	*erg*-mediated K^+^ current
*I*_K(M)_	M-type K^+^ current
*I*_Na(L)_	late Na^+^ current
*I*_Na(P)_	persistent Na^+^ current
*I*_Na(T)_	transient (peak) Na^+^ current
K_erg_ channel	*erg*-mediated K^+^ channel
K_M_ channel	M-type K^+^ channel
Na_V_ channel	voltage-gated Na^+^ channel
SGLT	Na^+^-dependent glucose co-transporter
V_ramp_	ramp voltage
